# Prescription patterns in psychiatric compulsory care: polypharmacy and high-dose antipsychotics

**DOI:** 10.1192/bjo.2021.982

**Published:** 2021-08-16

**Authors:** Katerina Kaikoushi, Maria Karanikola, Nicos Middleton, Evanthia Bella, Andreas Chatzittofis

**Affiliations:** School of Health Sciences, Department of Nursing, Cyprus University of Technology, Cyprus; and Cyprus mental Health Services, Famagusta, Cyprus; School of Health Sciences, Department of Nursing, Cyprus University of Technology, Cyprus; School of Health Sciences, Department of Nursing, Cyprus University of Technology, Cyprus; Medical School, University of Cyprus, Cyprus; Medical School, University of Cyprus, Cyprus; and Department of Clinical Sciences, Umeå University, Umeå, Sweden

**Keywords:** Polypharmacy, high-dose antipsychotics, compulsory, involuntary

## Abstract

**Background:**

Antipsychotic polypharmacy and prescription of high-dose antipsychotics are often used for the treatment of psychotic symptoms, especially in compulsory psychiatric care although there is lack of evidence to support this practice and related risks for patients.

**Aims:**

We aimed to investigate prescription patterns in patients with psychosis under compulsory psychiatric treatment in Cyprus and to identify predictors for pharmaceutic treatment patterns.

**Method:**

This was a nationwide, descriptive correlational study with cross-sectional comparisons, including 482 patients with compulsory admission to hospital. Sociodemographic and clinical data were collected. Psychotic symptoms were assessed with the Positive and Negative Syndrome Scale (PANSS). Prescribed medication patterns, including use of medication *pro re nata* (PRN, when required), were recorded.

**Results:**

Antipsychotic polypharmacy with a PRN schema was reported in 33.2% (*n* = 160) of the participants. Polypharmacy without a PRN schema was reported in 5.6% (*n* = 27) of the participants. We found that 27.2% (*n* = 131) of the participants were prescribed high-dose antipsychotics without PRN included; and 39.2% (*n* = 189) prescribed high-dose antipsychotics with PRN included. In the logistic regression analyses, predictors for prescription of high-dose antipsychotics were male gender, positive psychiatric history, receiving state benefits and a negative history of substance use. Male gender was the only predictor for polypharmacy without a PRN schema whereas male gender, negative family psychiatric history, receiving state benefits and the total score on the positive symptoms PANSS subscale were predictors for polypharmacy with a PRN schema included.

**Conclusions:**

A high frequency of polypharmacy and use of medication PRN beyond clinical guidelines has been reported for the first time in psychiatric compulsory care in Cyprus; revision in antipsychotic prescription is needed.

## Background

Management of psychotic symptoms is a challenging clinical task and there is data showing that published treatment guidelines are not always followed by clinicians.^1^ Research data regarding antipsychotic agents is mainly focused on monotherapy, effectiveness issues, optimisation of dosage, different routes of administration and pharmacokinetics, especially for long-acting medication.^2^ Monotherapy is not always achieved, and polypharmacy and prescription of high-dose antipsychotics are common practices in patients with psychotic symptoms.^2–4^

Antipsychotic polypharmacy is defined as prescribing more than one antipsychotic substance, and is usually justified for the treatment of resistant schizophrenia; in brief periods of medication cross-titration; and mainly when therapeutics include clozapine.^5^ However, in everyday clinical practice, treatment regimens may include patterns of use that are beyond these guidelines, with polypharmacy becoming a global practice with an approximately 20% frequency of occurrence.^3,6,7^ Several reasons have been cited, such as treatment-resistant illness, side-effects as well as the preferences of patients or physicians.^3,6,7^ Indeed, a combination of multiple antipsychotics with high doses of antipsychotics may be prescribed for the remission of symptoms.^8^

The practice of polypharmacy is, nevertheless, controversial, as there is scant data to support its effectiveness.^6^ In contrast, a wealth of empirical findings report risks arising from polypharmacy, including multiple side-effects, high mortality,^8^ high frequency of readmissions associated with an inability to follow complex pharmacotherapy patterns – all leading to high costs in healthcare systems.^9–11^

High-dose here is defined as when the total daily dose of an antipsychotic exceeds the upper dose limit recommended by clinical guidelines (for example those of the European Medication Agency or the British National Formulary) according to the patient's age and the indication being treated.^2^^,^^8^ Two practices related to prescription of high-dose antipsychotics have been identified. The first includes prescription of a single antipsychotic agent at a dose higher than the maximum recommended. The second practice regards the prescription of two or more antipsychotics, where the sum of the percentage of the maximum dosage of each agent corresponds to a total dose higher than 100%.^2,8^ As in the case of polypharmacy, prescribing high-dose antipsychotic medications is a practice that is not supported by research data and it is also associated with multiple risks, such as the occurrence of extrapyramidal symptoms and increased cardiac problems.^8,12,13^

Compulsory psychiatric treatment under involuntary admission to hospital is provided when individuals facing severe mental health problems refuse medical treatment and risk assessment about their or others’ safety, supports this practice. According to the Compulsory Psychiatric Hospitalisation Act of the Republic of Cyprus, involuntary psychiatric admission to hospital is based on a psychiatrist's clinical judgement when there is present a severe psychiatric disorder characterised by violent and harmful antisocial behaviour, or when the mental capacity of a patient has deteriorated to such an extent that it makes the patient's detention necessary for the protection of himself/herself and/or his/her beloved ones.^14^

Compulsory treatment has been associated with increased risk for both polypharmacy and prescription of high-dose antipsychotics. Thus, it is deemed important to monitor this practice to improve the quality and safety of care during compulsory treatment. Previous studies on patterns of antipsychotic prescription have not always included key variables, such as medication provided *pro re nata* (PRN, when required) and other clinical data, such as the severity of psychotic symptoms.^15,16^ PRN medication prescription is when timing of administration is left to the patient or clinician (nurse) as needed, or as the situation arises, in contrast to a scheduled medication plan.

## Aims

The aim of this study was to investigate prescription patterns in patients with psychosis involuntarily admitted to hospital for compulsory treatment in Cyprus, with a focus on polypharmacy and prescription of high-dose antipsychotics as well as identification of possible predictors for these treatment patterns. These patterns have not been evaluated in Cyprus previously; a situation which is mirrored in several other national contexts.^2–4^

## Method

### Study settings

This is a nationwide, descriptive correlational study with cross-sectional comparisons. The study took place at the Athalassa Psychiatric Hospital (APH), which is the only in-patient facility for compulsory psychiatric care in the Republic of Cyprus.

### Study participants and data collection

Data collection took place from December 2016 to February 2018 using a census sampling method. Following the inclusion and exclusion criteria, all demographic and clinical data of the study target population (i.e. adults involuntarily admitted with psychotic symptomatology to the APH), were recorded.

The inclusion criteria were:

- age between 18 to 65 years;
- diagnosis of a mood disorders, or substance use-induced disorder or schizophrenia-spectrum and other psychotic disorders;
- hospital admission of 3 days or longer;
- signed informed consent for participation in the study.

Exclusion criteria included diagnoses of:

- neurocognitive disorders, such as Alzheimer's disease or delirium;
- intellectual disabilities;
- developmental disorders; and
- personality disorders, as in most cases individuals with personality disorders were admitted to hospital for less than 24 h.

There was a total of 761 admissions to the APH during the study period. There were 22 individuals who were not included because of the age criterion (*n* = 9 were younger than 18 years; *n* = 13 were older than 65 years) and 173 were excluded because of their diagnosis (personality disorders *n* = 152; intellectual disabilities *n* = 21). Additionally, 77 individuals did not provide informed consent, 2 individuals died during their admission and 5 individuals were not included because of incomplete data. The final sample consisted of 482 cases.

### Assessment

Study participants were assessed independently by two members of the research team. The assessment took place within the first 72 h after admission with a duration of about 15–20 min. When participants’ native language was neither Greek nor English (4.3% of the participants), a translator supported the assessment process. Recorded assessment data were kept in patient's medical files, until signed informed consent was provided by them for inclusion in the present study. The informed consent was taken on the last day of hospital admission in order for the responders to have achieved an adequate level of illness insight, thus being able to fully understand the information given according to the research protocol. For the purposes of the analysis, only anonymised data were used.

A structured questionnaire was used for data collection, including the Positive and Negative Syndrome Scale (PANSS) for the assessment of the severity of psychotic symptoms,^17^ sociodemographic data (gender, age, marital status, nationality, educational level, occupational status, receiving financial reimbursement, body mass index (BMI)) and clinical data (personal and family psychiatric history, substance use, main symptomatology during admission, psychiatric diagnosis and prescribed medication).

Main symptomatology during admission was categorised as follows:

- non-adherence to pharmacotherapy and disorganised behaviour;
- substance use and disorganised behaviour;
- Suicidal/self-harming behaviour;
- aggressive behaviour towards others;
- disorganised behaviour not otherwise specified.

Psychiatric diagnoses were classified according to the following grouping:

- schizophrenia,
- other psychotic disorder from the spectrum of schizophrenia;
- mood disorders;
- other (anxiety disorders, psychotic disorders owing to a medical condition).

Regarding prescribed medication the following were recorded: active agent, total daily dose, route of administration, weekly dose of long-acting injectable (LAI) antipsychotics, active ingredients of other drug therapies, *statim* (STAT, immediately) and PRN schemas. As PRN prescription is a common practice and, in some cases, constitutes a large part of the prescribed medication schemas, it was deemed as important to assess its impact. Moreover, the prescribed dose of each antipsychotic was reported, and the equivalent percentage of the antipsychotic was calculated by converting its dose into the percentage of the maximum recommended daily dose for that agent according to the European Medicines Agency standards published in Maudsley prescribing guidelines.^18^ For example, since the maximum recommended daily dose of olanzapine is 20 mg, a dose of 10 mg corresponds to the 50% of the daily recommended antipsychotic dose. Moreover, the sum of all percentages prescribed to each patient was calculated. A ‘high-dose prescription’ was considered when the sum of all the percentages of the antipsychotics prescribed to a patient exceeded 100%. Antipsychotic polypharmacy was considered to be present when co-prescription of more than one antipsychotic was detailed for a patient.^19^

The study protocol was approved by the National Committee of Bioethics (EEBK/EP/2014/08), the Research Committee of the Ministry of Health (PN:5.34:01.7.3E) and the Personal Data Protection Officer (5.43.01.7.6 Ε, PN:0237/2014) of the Republic of Cyprus.

### Data analysis

Frequencies were used to describe categorical variables, and the chi-square test for comparisons between groups. Following normality tests for continuous variables, comparisons between groups were based on parametric tests. Moreover, age was analysed aa a categorical variable to provide more accurate information regarding specific age groups associated with polypharmacy and/or prescription of high-dose antipsychotics.

Prescribed medication were categorised first-/second-generation antipsychotics, LAI antipsychotics, antidepressants, mood stabilisers, anticholinergics (benzodiazepines and tranquilisers) and frequencies were estimated for each category. The association of clinical and sociodemographic characteristics with polypharmacy (with and without PRN schema) and prescription of high-dose antipsychotics (with and without PRN schema) were assessed using chi-square tests.

Statistical significance in univariable tests was set to 0.05 or lower. Aiming to explore these associations after controlling for the potential confounding effect of other variables, odds ratio (and 95% CIs) of polypharmacy and prescription of high-dose antipsychotics for each of the sociodemographic and clinical characteristics were estimated in logistic regression models. Forward-stepwise multivariable logistic regression models were used to select the final set of variables (among a large number) associated with high doses of antipsychotics and antipsychotic polypharmacy (dependent variables, respectively) controlling for the potential confounding effect of the rest of the variables in the final model. For the multivariable model, statistical significance was set to 0.10 or lower. Data analysis was performed using the Statistical Package for Social Sciences (SPSS, Inc, Chicago, IL version 20.00).

## Results

### Sociodemographic and clinical characteristics

The final sample included 65.3% (315) men and 34.6% (167) women, of whom 55% were in the age group 35–65 years. The majority were Greek Cypriots (74.5%), not married (86.9%) and unemployed (77.5%). Nearly half (48.8%) were diagnosed with schizophrenia and an additional 26.1% with other related psychotic disorders. A total of 74.9% had a personal psychiatric history, 59.3% had had previous hospital admissions for compulsory psychiatric treatment in the APH and 44% had a history for substance use. The sociodemographic and clinical characteristics of the study participants are presented in Table 1.

**Table 1:** Demographic and clinical characteristics (*n* = 482)

Variable	*n* (%)
Gender	
Male	315 (65.3)
Female	167 (34.6)
Age group	
18–34	217 (45.0)
35–65	265 (55.0)
Ethnicity	
Greek-Cypriot	359 (74.5)
Other	123 (25.5)
Marital status	
Married	63 (13.1)
Not married	419 (86.9)
Education level	
Primary school education	74 (15.4)
Lower secondary school education	92 (19.1)
Higher secondary school education	196 (40.7)
Tertiary education	120 (24.9)
Occupation	
Employed	109 (22.6)
Unemployed	373 (77.4)
Psychiatric diagnosis	
Schizophrenia	235 (48.8)
Other psychotic disorder from the spectrum of schizophrenia	126 (26.1)
Mood disorder	114 (23.7)
Other	7 (1.5)
Main symptomatology led to the current involuntary hospitalisation	
Non-adherence to pharmacotherapy & disorganised behaviour	260 (53.9)
Substance use and disorganised behaviour	110 (22.8)
Suicidal/Self-harming behaviour	51 (10.6)
Aggressive behaviour towards others	41 (8.5)
Disorganized behaviour not otherwise specified	20 (4.1)
Personal history of involuntary hospital admission	
First admission	196 (40.7)
Readmission	286 (59.3)
Personal history of mental health problems	
1st episode of a mental health problem	121 (25.1)
Positive history of mental health problems	361 (74.9)
Personal substance use history	
Yes	212 (44.0)
No	270 (56.0)

The total mean score on the PANSS was 100.94 (score range 41–186, s.d. = 25.82) on the overall scale. With a mean of 29.32 (scale range 7–46, s.d.= 9.17) on the positive symptoms subscale, 21.03 (scale range 7–46, s.d. = 9.34) on the negative symptoms subscale and 50.59 (score range 24–88, s.d. = 12.12) on the general symptoms subscale.

### Prescribed medication without including PRN

Antipsychotics were prescribed in 422 of the participants (87.6%). These were classified as first-generation antipsychotics (haloperidol, chlorpromazine, fluphenazine) and second-generation antipsychotics (olanzapine, amisulpride, risperidone, asenapine, ziprasidone, paliperidone), prescribed in 29.5% (*n* = 142) and 71.8% (*n* = 346) of participants, respectively.

The most frequent first-generation prescribed antipsychotics were haloperidol (23.2%) and zuclopenthixol (8.7%), whereas the most frequent second-generation prescribed antipsychotics were olanzapine (39.8%), risperidone (17.8%) and quetiapine (4.8%). Furthermore, the most frequent combination of antipsychotics included olanzapine and haloperidol (15.6%, *n* = 440).

LAI antipsychotics were prescribed in 18 participants (3.7%) and anticholinergics in 24.7% (*n* = 119) participants. Antidepressants were prescribed in 10.4% (*n* = 50) and mood stabilisers in 9.5% (*n* = 46) participants, with only 1 participant prescribed lithium. Benzodiazepines were prescribed in 53.5% (*n* = 258) of the participants and only 2.5% (*n* = 12) were prescribed tranquilisers (clomethiazole).

In 60 of the 482 participants, STAT medication was prescribed at the admission stage; antipsychotics were prescribed in 48 and benzodiazepines in 15 participants.

### PRN medication

PRN medication was prescribed in 98.1% (*n* = 473) of the participants. In 27 participants, besides PRN, no other medication was prescribed. The great majority of PRN agents were antipsychotics (in 440 participants) in combination with anticholinergics (in 438 participants). Benzodiazepines were prescribed in 351 participants and tranquillisers (clomethiazole) in 91 participants. Finally, promethazine was prescribed in 63.5% (*n* = 306) of the participants.

Prescribed pharmaceutic agents and PRN schemas according to the participants’ diagnoses, are reported in Table 2.

**Table 2:** Prescribed medication and *pro re nata* (PRN, when required) prescribed medication according to psychiatric diagnosis (*n* = 482)

Medication agent	Schizophrenia (%)	Psychotic disorders (%)	Mood disorders (%)	Other (%)	Total (%)
Prescribed medication					
Antipsychotics	213 (44.2)	113 (23.4)	91 (18.9)	5 (1.0)	422 (87.6)
1st generation antipsychotics	81 (16.8)	32 (6.6)	28 (5.8)	1 (0.2)	142 (29.5)
2nd generation antipsychotics	168 (34.9)	100 (20.7)	73 (15.1)	5 (1.0)	346 (71.8)
Depot antipsychotics	14 (2.9)	2 (0.4)	1 (0.2)	1 (0.2)	18 (3.7)
Clozapine	1 (0.2)	−	−	−	1 (0.2)
Bromocriptine	4 (0.8)	1 (0.2)	−	−	5 (1.0)
Antidepressants (SSRIs, SNRIs, NaSSA)	9 (1.9)	10 (2.1)	30 (6.2)	1 (0.2)	50 (10.4)
Mood stabilisers	17 (3.5)	10 (2.1)	18 (3.7)	1 (0.2)	46 (9.5)
Anticholinergics	80 (16.6)	22 (4.6)	17 (3.5)	−	119 (24.7)
Benzodiazepines	117 (24.3)	61 (12.7)	77 (16.0)	3 (0.6)	258 (53.5)
Tranquilisers/sleeping drugs	3 (0.6)	5 (1.0)	4 (0.8)	−	12 (2.5)
PRN prescribed medication					
Antipsychotics	224 (46.5)	117 (24.3)	95 (19.7)	4 (0.8)	440 (91.3)
Anticholinergics	225 (46.7)	114 (23.7)	95 (19.7)	4 (0.8)	438 (90.9)
Benzodiazepines	173 (35.9)	91 (18.9)	85 (17.6)	2 (0.4)	351 (72.8)
Tranquilisers/sleeping drugs	48 (10.0)	19 (3.9)	22 (4.6)	2 (0.4)	91 (18.9)
Promethazine	156 (32.4)	78 (16.2)	69 (14.3)	3 (0.6)	306 (63.5)

SSRIs, Selective Serotonin Reuptake Inhibitors; SNRIs, Serotonin and Norepinephrine Reuptake Inhibitors; NaSSA, Noradrenergic and Specific Serotonergic Antidepressant.

### Polypharmacy and prescription of high-dose antipsychotics

Only 5.6% (*n* = 27) of the participants were prescribed with more than one antipsychotic when PRN was not included (polypharmacy–PRN not included). When PRN was included (Polypharmacy–PRN included), polypharmacy rates increased to 33.2% (*n* = 160).

A total of 27.2% (*n* = 131) of the participants were classified as prescribed ‘high-dose antipsychotics–PRN not included’ (range 0–1520%, mean 157.44%, s.d. = 309.07%). When PRN was included in the therapeutic pattern, 39.2% (*n* = 189) of the participants were classified as prescribed ‘high-dose antipsychotics–PRN included’ (range 0–1640%, mean 194.81%, s.d. = 311.81%).

### Clinical characteristics of patients with polypharmacy and prescription of high-dose antipsychotics

Tables 3 and 4 present the sociodemographic and clinical characteristics in relation to polypharmacy and prescription of high-dose antipsychotics (with or without PRN included) in those prescribed antipsychotics (*n* = 440), respectively. Male gender (*P* = 0.0008) and low BMI (underweight status) (*P* = 0.014) were positively and statistically significantly associated with polypharmacy–PRN not included. Regarding polypharmacy–PRN included, positive associations were observed with male gender, low BMI (underweight status), Greek-Cypriot nationality, primary education only, unemployment status, positive history of compulsory hospital admissions and personal psychiatric history (all *P* < 0.05) (Table 3).

**Table 3:** Polypharmacy and sociodemographic and clinical characteristics in the study participants (*n*= 482)

	Polypharmacy							
	Without PRN included				With PRN included			
	% (*n*)	χ^2^	d.f.	*P*	% (*n*)	χ^2^	d.f.	*P*
Gender								
Male	7.8 (24)	6.99	1	0.008	39.7 (125)	17.25	1	<0.001
Female	1.8 (3)				21.0 (35)			
Age								
18–34 years old	6.5 (14)				33.2 (72)			
35–65 years old	4.9 (13)	0.539	1	0.463	33.2 (88)	0.00	1	0.995
BMI								
Low (underweight)	0.2 (1)	6.04	1	0.014	0.5 (2)	6.04	1	0.008
Normal	2.6 (11)				15.1 (65)			
High (overweight)	1.9 (8)				8.4 (36)			
Very high (obese)	0.9 (4)				8.4 (36)	5.10	1	0.018
Nationality								
Greek-Cypriot	6.4 (22)	0.737	1	0.390	36.5 (131)	6.88	1	0.009
Other	4.1 (5)				23.6 (29)			
Marital status								
Married	4.8 (3)				31.7 (20)	0.07	1	0.793
Unmarried	5.7 (24)	0.097	1	0.756	33.4 (140)			
Educational attainment								
Primary	10.8 (8)	4.68	3	0.200	43.2 (32)	0.73	1	0.044
Lower secondary	5.4 (5)				38.0 (35)			
Higher secondary	4.6 (9)				32.1 (63)			
Tertiary	4.2 (5)				25.0 (30)			
Occupational status								
Employed	4.6 (5)				23.9 (26)			
Unemployed	5.9 (22)	0.274	1	0.601	35.9 (134)	5.54	1	0.019
Psychiatric diagnosis								
Schizophrenia	6.0 (14)	2.89	3	0.409	38.3 (90)	6.41	3	0.093
Other psychotic disorder	3.2 (4)				25.4 (32)			
Mood disorder	7.0 (8)				31.6 (36)			
Other	14.3 (1)				28.6 (2)			
Main symptomatology that led to current involuntary hospital admission								
Non-adherence to pharmacotherapy and disorganised behaviour	10.0 (17)	3.22	4	0.521	35.8 (93)	4.95	4	0.292
Substance use and disorganised behaviour	2.4 (6)				32.7 (36)			
Suicidal/self-harming behaviour	2.0 (1)				35.3 (18)			
Aggressive behaviour towards others	5.5 (1)				19.5 (8)			
Disorganised behaviour not otherwise specified	6.5 (2)				25 (5)			
Personal history of involuntary hospital admission								
First admission	5.1 (10)				24.5 (48)			
Readmission	5.9 (17)	0.156	1	0.693	39.2 (112)	11.28	1	0.001
Personal history of mental health problems								
First episode	2.5 (3)				23.1 (28)			
Prior history	6.6 (24)	2.97	1	0.084	36.6 (132)	7.36	1	0.007
Personal history of substance use								
Yes	2.5 (12)				34.9 (74)			
No	3.1 (15)	0.002	1	0.960	31.9 (86)	0.499	1	0.480

PRN, *pro re nata* (when required).

**Table 4:** High-dose antipsychotics and sociodemographic and clinical characteristics in the study participants (*n* = 482)

	High doses of antipsychotics							
	Without PRN included				With PRN included			
	% (*n*)	χ^2^	d.f.	*P*	% (*n*)	χ^2^	d.f.	*P*
Gender								
Male	30.2 (95)	4.08	1	0.043	45.4 (143)	14.59	1	<0.001
Female	21.6 (36)				27.5 (46)			
Age								
18–34 years old	22.6 (49)				35.9 (78)			
35–65 years old	30.9 (82)	4.21	1	0.04	41.9 (111)	1.76	1	0.184
BMI								
Low (underweight)	0.7 (3)	3.81	1	0.035	0.5 (2)	6.32	1	0.008
Normal	16.2 (70)	3.03	1	0.050	10.4 (45)	4.22	1	0.025
High (overweight)	11.4 (49)				7.7 (33)			
Very high (obese)	9.5 (41)	7.00	1	0.007	7.4 (32)	4.82	1	0.020
Nationality								
Greek-Cypriot	30.1 (108)	5.99	1	0.014	42.6 (153)	6.85	1	0.006
Other	18.7 (23)				29.3 (36)			
Marital status								
Married	4.8 (3)				31.7 (20)			
Unmarried	5.7 (24)	0.071	1	0.790	33.4 (140)	0.083	1	0.846
Educational attainment								
Primary	41.9 (31)	23.19	3	0.002	59.5 (44)	14.62	3	<0.0001
Lower secondary	33.7 (31)				47.8 (44)			
Higher secondary	21.9 (43)				33.7 (66)			
Tertiary	21.7 (26)				29.2 (35)			
Occupational status								
Employed	19.3 (21)				6.8 (33)			
Unemployed	29.5 (100)	4.45	1	0.035	32.4 (156)	4.73	1	0.003
Psychiatric diagnosis								
Schizophrenia	32.8 (77)	9.48	3	0.023	46.0 (108)	10.77	3	0.013
Other psychotic disorder	19.0 (24)				28.6 (36)			
Mood disorder	23.7 (27)				36.8 (42)			
Other	42.9 (3)				42.9 (3)			
Main symptomatology which led to current involuntary hospital admission								
Non-adherence to pharmacotherapy and disorganised behaviour	31.2 (81)	6.43	4	0.169	43.1 (112)	8.58	4	0.072
Substance use and disorganised behaviour	21.8 (24)				38.2 (42)			
Suicidal/self-harming behaviour	29.4 (15)				41.2 (21)			
Aggressive behaviour towards others	17.1 (7)				22.0 (9)			
Disorganised behaviour not otherwise specified	20.0 (4)				25.0 (5)			
Personal history of involuntary hospital admission								
First admission	17.9 (35)				27.0 (53)			
Readmission	36.6 (96)	11.28	1	<0.0001	47.6 (136)	20.52	1	<0.0001
Personal history of mental health problems								
First episode	14.0 (17)				24.0 (29)			
Prior history	31.6 (114)	14.50	1	<0.0001	44.3 (160)	15.75	1	<0.0001
Personal history of substance use								
Yes	21.7 (46)				38.7 (81)			
No	31.5 (85)	5.73	1	0.017	39.6 (107)	0.045	1	0.832

PRN, *pro re nata* (when required).

Male gender, age between 35 and 65 years, low BMI (underweight status), Greek-Cypriot nationality, primary education only, unemployment status, diagnosis of schizophrenia, previous compulsory hospital admission, personal psychiatric history and positive history of substance use were all significantly positively associated with the prescription of high-dose antipsychotics– PRN not included (all *P* < 0.05). Male gender, age between 35 and 65 years, low BMI (underweight status), Greek-Cypriot nationality, primary education only, unemployment status, diagnosis of schizophrenia, previous compulsory hospital admission and personal psychiatric history were all significantly positively associated with prescription of high-dose antipsychotics– PRN included (all *P* < 0.05) (Table 4).

### Association of sociodemographic and clinical characteristics with polypharmacy and prescription of high-dose antipsychotics

Table 5 presents the predictors of polypharmacy (with and without PRN included) and prescription of high-dose antipsychotics (with and without PRN included) as estimated in multivariable stepwise logistic regression analyses. Positive personal psychiatric history, male gender, receiving state benefits and negative history of substance use were associated with prescription of high-dose antipsychotics, both with and without PRN included. For instance, men were 2.2–2.6 times more likely to be prescribed with high-dose antipsychotics, without and with PRN included, even after accounting for the other variables in the model.

**Table 5:** Adjusted odds ratios (and 95% CI) of high doses of antipsychotics and polypharmacy, with and without *pro re nata* (PRN) medication included by clinical and sociodemographic characteristics, as estimated in multivariable stepwise logistic regression models (*n* = 440)

	Without PRN included			With PRN included		
	aOR	95% CI	*P*	aOR	95% CI	*P*
High dose of antipsychotics						
Personal history of mental health problems – not first episode	2.158	1.203−3.872	0.010	1.782	1.083−2.932	0.023
Gender – male	2.175	1.312−3.606	0.003	2.585	1.590−4.203	<0.001
Financial allowance – yes	2.225	1.430−3.461	<0.001	1.783	1.182−2.689	0.006
History of substance use – yes	0.427	0.265−0.688	<0.001	0.631	0.406−0.981	0.041
Educational attainment – primary (versus tertiary)	–	–	–	2.322	1.297−4.159	0.005
Polypharmacy						
Gender – male	4.484	1.330−15.122	0.019	2.204	1.379−3.524	0.001
Family psychiatric history – no	–	–	–	2.106	1.146–3.868	0.016
Receiving state benefits – yes	–	–	–	1.803	1.201−2.707	0.004
Total score of positive symptoms PANSS subscale, per unit increase	–	–	–	1.049	1.024−1.075	<0.001

aOR, adjusted odds ratio; PANSS, Positive and Negative Syndrome Scale.

Primary education only was also a predictor of prescription of high-dose antipsychotics with PRN included, with an adjusted odds ratio (OR) of 2.3 (95% CI 1.3–4.2) compared with people with tertiary education. Male gender was the only predictor of polypharmacy with PRN not included. When PRN was included, male gender, negative family psychiatric history, receiving state benefits and total score on the PANSS positive symptoms subscale were all statistically significantly associated with an increased likelihood of polypharmacy (Table 5). In terms of the PANSS positive symptoms subscale, there appeared to be a 1.049 (95% CI 1.024−1075) higher likelihood of polypharmacy with PRN included per unit increase in PANSS score.

## Discussion

### Main findings

In this nationwide study, medication prescription patterns were investigated in patients under compulsory psychiatric care; with approximately one out of three (33.2%) meeting the criteria for polypharmacy and 39.2% of them meeting the criterion for prescription high-dose antipsychotics (including PRN). This is the first study in the Republic of Cyprus exploring prescription patterns of antipsychotics.

### Comparison with findings from other studies

The findings are in line with the literature reporting that although efforts have been made through guidelines to avoid prescription of high-dose antipsychotics and polypharmacy,^20,21^ this remains a common practice. Compulsory psychiatric treatment has been associated with both polypharmacy and prescription of high-dose antipsychotics in several studies.^2,5,22^

The present study suggest that polypharmacy is because of the use of medications PRN; mainly haloperidol, almost universally prescribed to all patients. This may suggest that the use of PRN medication is mainly targeted at sedation and management of dysfunctional behaviours in in-patients (such as agitation, violent reactions) rather than having a therapeutic effect.^2^ Similar findings were observed in the study by Mendes et al,^2^ where approximately half of the participants were prescribed with ‘as required’ antipsychotics. Nevertheless, this issue deserves more attention in future research.

In modern psychiatry, striving for patient-centred care, the practice of ‘one size fits all’ seems to be established in the case of Cyprus, a pharmacotherapy pattern that needs to be further investigated. Nevertheless, the rate of polypharmacy without a PRN schema was 5.6%, which appears to be among the lowest reported in the literature. Polypharmacy rates range widely across different countries; for instance, a rate of around 17% has been reported in the UK and Australia,^9,23^ 32% in Italy^24^ and 41.6–70.5% in Portugal.^2,4^

### Interpretation of our findings

A systematic review and meta-analysis of 147 studies on polypharmacy reported that the median rate of antipsychotic polypharmacy was 19.6%.^3^ Polypharmacy in individuals with mental health problems has been associated with diagnoses of severe mental disorders, including psychosis.^25^ As expected, this diagnosis is common in patients involving involuntary hospital admissions, along with limited response to medication treatment and inadequate insight about disease;^26^ characteristics that were also present among participants in the present study. Indeed, polypharmacy was positively associated with the PANSS positive symptoms subscale score. The present findings also demonstrated that polypharmacy was associated with male gender, positive personal psychiatric history, receiving state benefits, positive history of involuntary hospital admissions and positive history of substance use. These factors have been previously associated with a worse prognosis in patients with psychosis.^27^

Moreover, the reported low rate of polypharmacy when PRN schema was not included (5.6%), may imply that PRN schemas are not used as indicated in published guidelines (i.e. as circumstances require),^1,18^ but rather as part of the formal pharmacotherapy plan. Additionally, such an extensive use of PRN schemas may suggest that these are not primarily used for their therapeutic effect but as a form of chemical restraint towards aggressiveness during hospital admissions; a practice previously reported.^2^ Nevertheless, there is no robust evidence that PRN use in patients with psychosis is effective or even beneficial; a recent review reported lack of randomised controlled trials on the subject, or any other evidence to support this practice.^15^ In fact, the review underlined caution on PRN use and highlighted patients’ consent on this, a status compromised in compulsory psychiatric care.

Furthermore, the present findings reported a rate of 27.2% of prescription of high-dose antipsychotics–PRN not included; that increased to 39.2% when PRN schemas were included. These rates are much higher compared with the East Asia region (6.5%)^26^ and other countries, such as the UK (6.8%),^9,24^ Australia (20.5%)^5^ and the Netherlands (25.5%).^28^ Only two studies, one German and one Portuguese, have reported higher rates of prescription of high-dose antipsychotics (42.5%, 51.4%, respectively),^4,28^ as the results of the present study show; and in another study in Portugal, prescription of high-dose antipsychotics was 13.8% when PRN was not included, and 49.2% when PRN was included.^2^ Overall, a high frequency of prescription of high-dose antipsychotics may also support the hypothesis that PRN schemas are mainly used as a means of chemical restraint, unfortunately leading to high total daily doses of antipsychotics, compromising quality and patient safety.

In this study, those unemployed and receiving state benefits, and with primary education only appeared to be more likely to be prescribed with high-dose antipsychotics, as previously reported.^2^ This may imply that this group of patients is either inadequately socially adjusted or supported, and/or is presenting with more severe symptomatology, probably as a result of low adherence to therapy. Data show that those with stable social support systems and higher education report increased medication adherence; thus, they are less prone to relapse and being prescribed with high-dose antipsychotics and involuntary hospital admissions for compulsory treatment.^29^ Moreover, one may argue that clinicians may rely on enhanced medication schemas in the absence of a functional social network, or effective community mental health services.

Individuals with a negative history of substance use had a lower risk being prescribed high-dose antipsychotics. This might be explained by the fact that, substance-induced psychotic symptoms most of the times decline rapidly; thus, antipsychotic medication is not titrated to high dosage.^30^

Individuals with a positive history of involuntary psychiatric hospital admissions were more frequently prescribed high-dose antipsychotics. Indeed, a history of previous compulsory psychiatric treatments suggests a longer disease duration, which usually requires higher doses of antipsychotics to manage symptoms adequately.^11^ The finding that those participants with a negative family history were more frequently prescribed two or more antipsychotics may be explained by a delayed approach to mental health services because of a lack of mental health literacy and knowledge, or stigma;^31^ it is expected that those who are more familiar with psychiatric symptoms may reach mental health services earlier if they experience relevant symptoms. Nevertheless, there might be additional confounding factors not explored, such as duration of untreated psychosis or age of onset.^31^

### Findings relating to the type of antipsychotics prescribed

The most frequently prescribed antipsychotics herein were olanzapine, risperidone, quetiapine, haloperidol and zuclopenthixol. Similarly, previous studies have shown that olanzapine, risperidone and quetiapine are the most frequently prescribed second-generation antipsychotics,^32^ whereas haloperidol^4^ and zuclopenthixol^2^ are the most frequently prescribed first-generation antipsychotics^2,4^ in those involuntarily admitted for psychiatric treatment. Moreover, in line with the present results, previous data show that the most frequent combination of antipsychotics included one first-generation antipsychotic and one second-generation antipsychotic.^2^

### Findings relating to BMI

The association of low BMI with prescription of high-dose antipsychotics in this study is in contrast with previous data showing a positive association between increased BMI and prescription of high-dose antipsychotics;^33^ this relationship was explained on the basis of weight gain as a side-effect of increased doses of antipsychotics.^34^ The relationship needs to be further explored.

### Findings relating to LAI and clozapine

There were additional interesting findings in the present study, such as under-prescription of LAI antipsychotics (3.7%); yet this is in line with the guidelines for the prescription of LAI antipsychotics (except monthly administration of paliperidone), which are licensed for maintenance therapy instead of controlling acute psychiatric conditions. Moreover, use of LAI antipsychotics require an adequate trial of oral therapy before administration.^35^

Only one patient was prescribed clozapine, an antipsychotic with an indication for the treatment of resistant schizophrenia. This might be explained by the fact that clozapine requires a strict initiation and titration protocol for administration, thus making it difficult to be initiated within the first 72 h after involuntary hospital admission for compulsory treatment.^36^ In contrast, data on community treatment orders, as a form of compulsory treatment of individuals diagnosed with a mental illness in the community, show that first-generation LAI antipsychotics and clozapine are associated with antipsychotic polypharmacy, and oral second-generation antipsychotics and LAI risperidone are both associated with prescription of high-dose antipsychotics.^37^

### Implications

Recommendations derived from this study may include the revision of the PRN practice in psychiatric compulsory care, especially in relation to haloperidol and its combination with anticholinergics, as well as following international guidelines.^38^ Indeed, a review regarding PRN practice revealed a lack of documentation on PRN prescription as well as relevant adverse reactions.^39^ Tools for decision-making and national guidelines for PRN administration including monitoring are also needed, as well as education and training of healthcare professionals. Furthermore, investment in depot and clozapine clinics in the community may contribute to a decrease in relapse rates, and therefore of involuntary hospital admissions in people with psychotic symptoms; individuals who are prescribed with LAI antipsychotics and clozapine seems to have lower relapse rates.^1,5,18^ Additionally, non-pharmacological strategies, such as verbal and non-verbal de-escalation of aggressiveness, along with relevant education, should be provided.^27,39^

Polypharmacy results in risks with adherence to therapy because of the complexity of medication schemas.^6^ The goal is to identify the lowest therapeutic dose of medication to minimise side-effects without compromising effectiveness in terms of relapse prevention.^11,12^ Finally, the universal prophylactic use of anticholinergic medication in patients treated with antipsychotics needs to be further researched.

### Strengths and limitations

The most important strength of this study was the examination of prescription patterns in a relatively large census sample of patients under compulsory treatment in the referral in-patient facility in Cyprus. Moreover, this is the first study nationally to describe prescription patterns with a focus on antipsychotics and hence it provides a much-needed baseline time point for future assessment of progress. Important demographic and clinical variables including diagnostics and the assessment of psychotic symptoms were taken into consideration, which is an additional strength of the present study.

Limitations include the cross-sectional design and lack of data regarding cross-titration during switching, length of hospital admissions, the pharmacotherapy relating to the management of side-effects and other practices justifying polypharmacy such as short-term use of an oral antipsychotic when starting a LAI. Additionally, information on previous clozapine use, which would justify polypharmacy, or high doses of antipsychotics, was not available.

It should be noted that during the study several pharmaceutic agents were not available at APH, such as the relatively new LAI antipsychotics (olanzapine, paliperidone, aripiprazole), a situation that may have influenced prescription patterns. An additional limitation regards the categorisation of the main symptomatology during participants admission. However, the categorisation applied was in line with the documentation applied in acute mental health services in Cyprus. Moreover, the fact that the length of involuntary hospital admission has not been recorded is an additional limitation. Future studies need to address this variable as well.

In conclusion, the present findings demonstrate suboptimal practices relating to antipsychotic prescription in the most vulnerable psychiatric patients, i.e. those under compulsory psychiatry care. Specifically, we described a high frequency of polypharmacy and PRN schemas, which are not consistent with clinical guidelines. These provide evidence of the need to revise antipsychotic prescription practices, aiming to improve safety and quality of care. The goal is to shift from antipsychotic polypharmacy to monotherapy without affecting either psychiatric symptoms severity or the frequency of relapse.

## Data Availability

No data are available. Not applicable.
